# Oral and dental health status in patients with eating disorders in Madrid, Spain

**DOI:** 10.4317/medoral.23010

**Published:** 2019-08-19

**Authors:** Pablo Garrido-Martínez, Adelaida Domínguez-Gordillo, Rocío Cerero-Lapiedra, Miguel Burgueño-García, María-José Martínez-Ramírez, Carmen Gómez-Candela, José-Luis Cebrián-Carretero, Germán Esparza-Gómez

**Affiliations:** 1 DDS, PhD. Department of Bucofacial Medicine and Surgery, Faculty of Odontology, University Complutense of Madrid, Madrid, Spain; 2 DDS, PhD. Department of Preventive Medicine, Public Health and History of the Science, Faculty of Medicine, University Complutense of Madrid, Madrid, Spain; 3 MD, PhD. Department of Bucofacial Medicine and Surgery, Faculty of Odontology, University Complutense of Madrid, Madrid, Spain; 4 MD, PhD. Department of Oral and Maxillofacial Surgery, Hospital Universitario La Paz, Madrid, Spain; 5 MD, PhD. Department of Endocrinology and Nutrition, Hospital Universitario Jaén, Spain; 6 MD, PhD. Department of Endocrinology and Nutrition, Hospital Universitario La Paz, Madrid, Spain; 7 MD,DDS, PhD. Department of Oral and Maxillofacial Surgery, Hospital Universitario La Paz, Madrid, Spain; 8 MD, PhD. Senior Professor. Department of Bucofacial Medicine and Surgery, Faculty of Odontology, University Complutense of Madrid, Madrid, Spain

## Abstract

**Background:**

The aim of the present study was to describe and compare the oral and dental health status of two groups, one diagnosed with eating disorders (EDs), and another group without this pathology, assessing the following oral manifestations: dental alterations, periodontal disorders, soft tissue disorders, non-stimulated salivary flow, and oral pH.

**Material and Methods:**

This comparative transversal epidemiological study included 179 participants, of whom 59 were diagnosed with EDs (Eating Disorder Group: EDG) and 120 had no antecedents of EDs (No Eating Disorder Group: NEDG). All patients fulfilled the following inclusion criteria: women aged over 18 years, diagnosed with an ED by a specialist, patients who had undergone at least 1 year monitoring by the Clinical Nutrition Unit, and had not received any periodontal treatment during the previous 6 months. Both groups were homogeneous in terms of sex, age, education, and socioeconomic level. Oral exploration was performed, registering clinical variables, as well as sociodemographic and socioeconomic data, oral hygiene habits, and smoking. Statistical significance was established as *p*<0.05 (confidence level > 95%).

**Results:**

The dental erosion (DE) was the most significative feature of dental alterations. The degree of DE was significantly greater in the EDG (*p*<0.001). A significant association between soft tissue lesions and EDs was found (*p*<0.001) A notable difference in non-stimulated salivary flow was found between the groups (*p*<0.001). No significant differences between the groups were found for periodontal status, dental caries, or oral hygiene practices.

**Conclusions:**

On the basis of the results obtained, it is necessary to carry out oral/dental examination as soon as an ED is diagnosed with regular check-ups thereafter.

** Key words:**Eating disorders, anorexia nervosa, bulimia nervosa, oral health, dental erosion.

## Introduction

Eating disorders involve serious disturbances in eating behavior, such as extreme and unhealthy reduction of food intake or severe overeating, as well as feelings of distress or extreme concern about body shape or weight. The four most common eating disorders are Anorexia Nervosa (AN), Bulimia Nervosa (BN), Binge Eating Disorder (BED), and Other Specified Feeding or Eating Disorders (OSFED) following the diagnostic and statistical manual of mental disorders (DSM-5, 2013) (1).

AN is relatively common among young women. Recent years have seen an increase in the global incidence within the risk group: teenage girls aged between 15 and 19 years. However, BN has decreased since the nineteen nineties. All eating disorders present a high risk of mortality, especially AN. In comparison with other eating disorders, binge eating disorder (BED) is more common among men and older people (2).

The prevalence of eating disorders in adult women in six European countries was 0.9% for the AN, 2.3% for the BN and 1.9% for the OSFED (3). In Spain (4) , epidemiological data shows a prevalence in women between 3,71% - 5,46%, being OSFED the most frequent reported.

The behavioral disorders that accompany EDs involve certain habits that may have repercussions in the oral cavity either as direct damage or through a deficit in basic nutrition: dental erosion; periodontal disorders, such as gingivitis and periodontitis; dental caries; soft tissue disorders, such as erythema and ulcers; xerostomia; and changes in oral pH (5,6).

It has been shown that oral lesions are significantly associated with the frequency of self-induced vomiting (7,8). Diet and standard of living are other fundamental factors affecting the appearance of some oral pathologies in both ED patients and healthy subjects.

Early detection of these signs by dental personnel is fundamental in order to avoid the early development of irreversible lesions such as dental caries and dental erosion (9). Diagnosis can also contribute to early identification of patients with EDs (10).

Although numerous studies have been published previously with epidemiological data, only one has evaluated the oral manifestations produced among ED patients in Spain (11) but the patient sample was small. Data on the prevalence of oral disorders in other countries around the world is not homogeneous, with marked differences between different studies (12,13).

The aim of the present study was to describe and compare the oral and dental health status of two groups, one diagnosed with EDs, and another group without the pathology, assessing the following oral manifestations: DE, periodontal disorders, dental caries, soft tissue disorders, non-stimulated salivary flow, and oral pH.

## Material and Methods

Study design and population 

This transversal epidemiological study included 179 participants, of whom 59 were diagnosed with EDs and 120 had no antecedents of EDs.

The sample was recruited over 12 months (June 2014 to June 2015) from a population in the rural area covered by the La Paz University Hospital (UH) in Madrid, Spain. The ED group (EDG) consisted of patients diagnosed with an ED attending the hospital’s Clinical Nutrition Unit (Endocrinology and Nutrition Service). All patients fulfilled the following inclusion criteria: women aged over 18 years, diagnosed with an ED by a specialist, patients who had undergone at least 1 year monitoring by the Clinical Nutrition Unit, and had not received any periodontal treatment during the previous 6 months.

The no ED group (NEDG) was recruited from the Oral and Maxillofacial Surgery Unit, having attended a weekly check-up following third molar extraction. They were included in a non-probabilistic sample of consecutive cases, who were willing to take part in the study and fulfilled the same inclusion criteria as the EDG, with the exception of ED diagnosis, and matching them by age, educational and socioeconomic level.

Similar exclusion criteria were applied to both groups: patients undergoing dental treatment involving rehabilitation of dental structures that might impede evaluation of the degree of dental erosion, and/or diagnosed with some other major chronic disease involving malnutrition and bone metabolism and/or protein metabolism.

The study followed guidelines established in the Helsinki Declaration, and was approved by the Research Ethics Committee of the La Paz University Hospital (PI-1500). All participants volunteered and gave their informed consent to take part in the study. All data were treated in dissociated manner at all times, and fulfilled current data protection regulations in Spain.

-Data collection and variables

**Figure 1:**
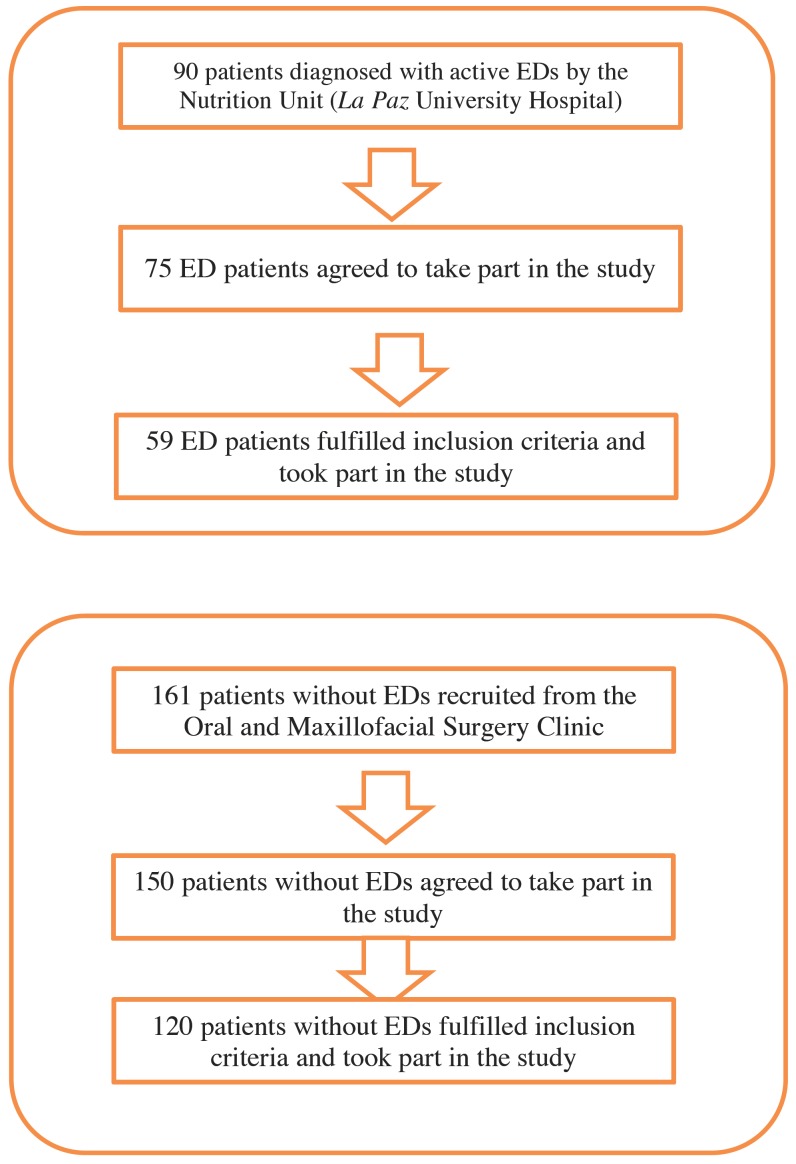
Flow diagram.

Each patient was called for oral exploration from the healthcare service she was attending: the Nutrition Unit, or Maxillofacial Surgery Clinic (Fig. 1).

Oral exploration was performed with the patient in a dental chair, which was equipped with all necessary materials and instruments, taking appropriate hygiene measures.

Oral exploration was performed by the same dentist, an expert in the diagnosis of the pathologies under evaluation, who was previously calibrated with an excellent intra-observer kappa statistic (Fleiss’ kappa).

During exploration, all clinical variables were registered, as well as sociodemographic and socioeconomic data, oral hygiene habits, and smoking.

At this point, the medical antecedents of patients remained unknown; this information was obtained later. These data were obtained from hospital medical records. In this way, the examiner was blinded at the time of oral exploration.

-The variables analyzed were.

-Sociodemographic variables: age (years); socioeconomic level and educational level.

-The oral hygiene habits registered were: frequency of dental check-ups (months), frequency of toothbrushing (number of times per day), frequency of mouthwash use and dental flossing (daily and weekly). Patients’ smoking habits were also recorded (number of cigarettes per day: evaluated as 0, fewer than 10, more than 10), and medication consumption.

-Dental variables analyzed and the methods used were as follows.

1. The degree of DE was measured using the technique described by Johansson *et al.* (14) 

2. The Ramfjord Periodontal Index (PI) (15) was use to evaluate periodontal status, using the WHO periodontal probe.

3. The presence of caries was determined visually using a dental explorer probe and dental mirror. Third molars were not included in exploration. Caries was evaluated using the decay-missing-filled (DMF) index, and restoration index (RI) (16).

4. Diagnosis of soft tissue lesions was performed clinically, using exploration probes, mirror and gauzes, and classified as present or absent. Any lesions present were recorded in photographs.

5. Non-stimulated salivary flow measurements were made using the draining technique to determine the flow rate expressed as ml/min for 5 minutes (17). The results were classified in terms of their clinical relevance as: normal salivary flow (>0.3ml/min), reduced (≤0.3ml/min - ≥0.1ml/min) and hyposialia (<0.1ml/min).

6. Salivary pH was evaluated as a quantitative variable, using pH Test Strips® (SIGMA Chemical Company, St. Louis, Mo USA) placed in the test tube used for saliva flow measurement.

-Statistical analysis

Descriptive statistics were calculated for quantitative variables (mean, standard deviation, maximum and minimum values). For qualitative variables, the distribution of absolute frequencies and percentages was determined for the whole sample and for the two groups. The chi-squared test and the Fisher exact test were applied to analyze associations between qualitative variables; for quantitative variables Student’s t-test was used, and ANOVA when necessary. Statistical significance was established as *p*<0.05 (confidence level > 95%). Standardized effect size measures were calculated through the Pearson correlation coefficient and Cramer’s V.

## Results

Both groups were homogeneous in terms of sex, age, education, and socioeconomic level.

The average age of the whole sample was 27.62 years, ranging from 19 to 44 years, without statistically significant differences between the groups.

Participants who had completed University education were the largest group. Regarding participants’ socio-economic levels, distribution was very similar between the two groups, with most patients’ families belonging to high socioeconomic groups (salaried managerial posts, individuals with higher education qualifications, sportsmen/women and creatives).

No differences were found between groups in the frequency of dental check-ups or oral hygiene habits. Approximately 90% of the sample performed toothbrushing two or more times per day. Half the sample used mouthwashes and 30% used dental floss ( Table 1).

**Table 1:**
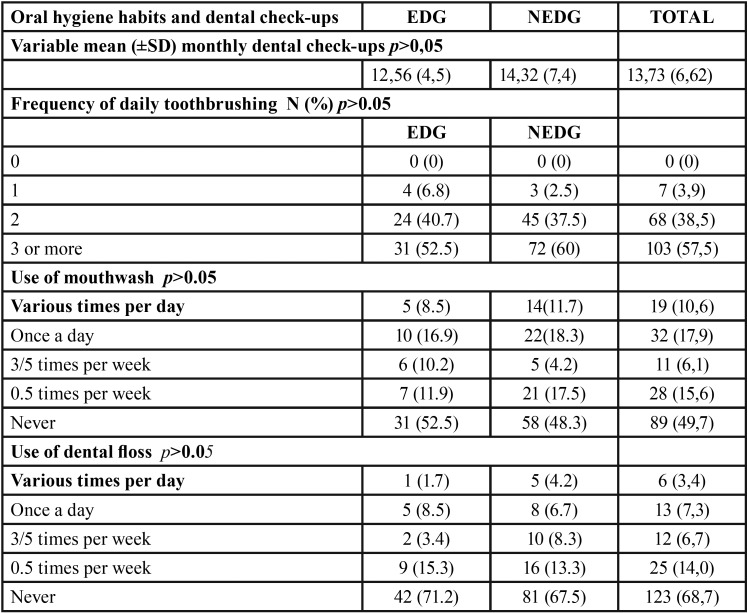
Oral hygiene habits and dental check ups.

In the present study, only 25.7% of the patients were smokers, smoking a mean of 6.54 cigarettes per day. In the EDG, the percentage increased to 37.3% compared with 20% in the NEDG (*p*=0013; Cramér’s V = 0.186).

EDG patients were classified according to the DSM–5 ( Table 2).

**Table 2:**
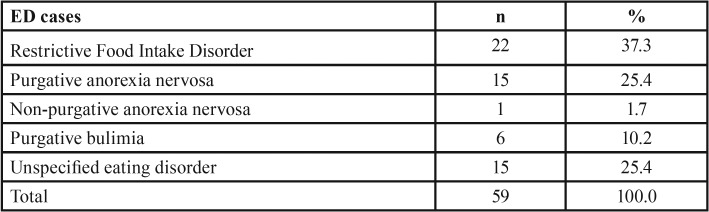
Classification of ED cases according to DSM-5.

 Table 3 shows the results for all dental/oral variables. The periodontal status and mean of caries were similar in both groups and no stadistical significances were found. A marked difference in non-stimulated salivary flow was found between the EDG and NEDG groups (*p*<0.001). Of the 59 EDG participants, only 28.8% presented a normal salivary flow; the other participants showed a reduced flow or hyposialia, while all NEDG participants, with one exception, presented a normal salivary flow. Measuring non-stimulated saliva flow, the EDG obtained a mean of 0.23 ml/min, a considerable reduction compared with the normal flow rate. In the NEDG, the average was 0.61 ml/min, (*p*<0.001). Twelve patients in the EDG (20.3%) obtained a non-stimulated flow of less than 0.1 ml/min, considered to represent hyposialia. Mean pH was found to be slightly higher in the EDG than in the NEDG but with no remarkable differences.

**Table 3:**
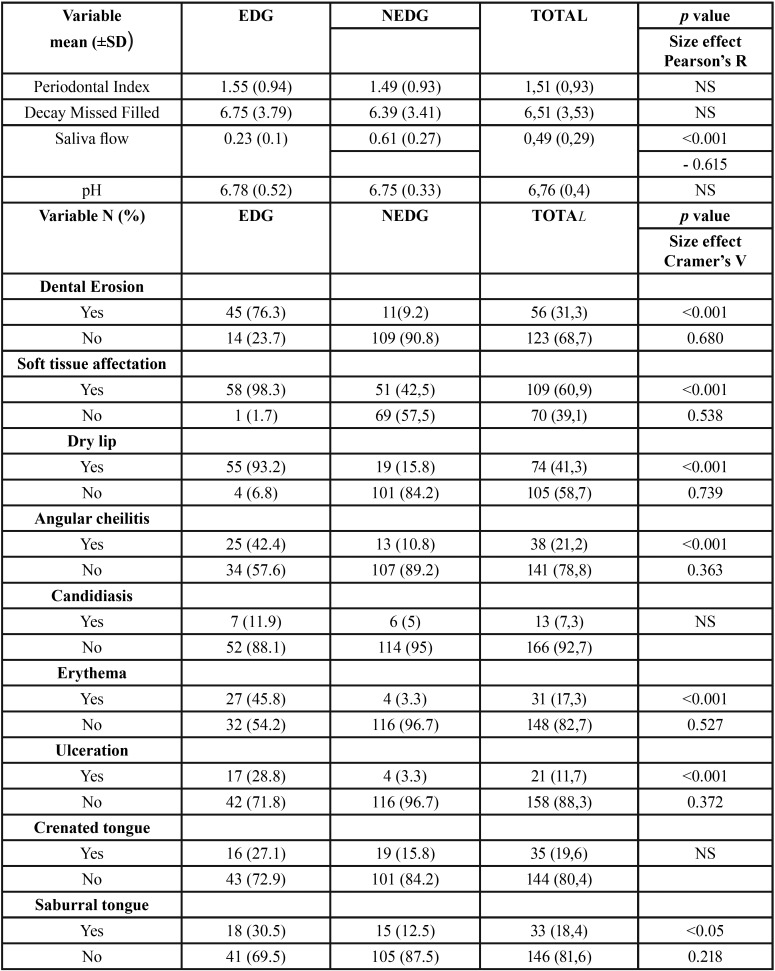
Dental/oral variables, means and differences between groups.

A significant association between soft tissue lesions and EDs was found (*p*<0.001), so that the EDG presented more soft tissue affectation (over 98%) than the NEDG (42.5%). Of all soft tissue lesions, were found statistically significant differences in dry lip, angular cheilitis, erythema, ulcerations (*p*<0,001) and saburral tongue (*p*<0,05) ( Table 3). In patients who smoked (both in the EDG and NEDG groups) a higher presence of soft tissue lesions was observed with statistical significance ( Table 4) (*p*<0.05).

**Table 4:**
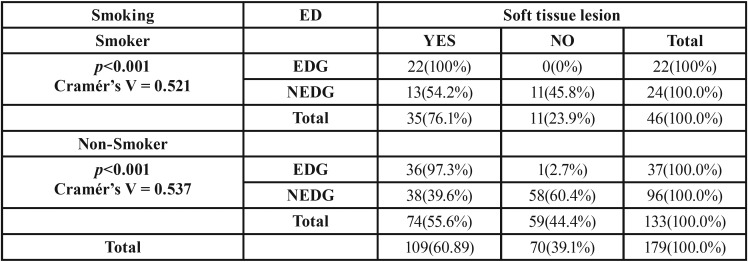
Association between smoking and soft tissue lesions.

Presence of DE was significantly greater in the EDG than the NEDG (*p*<0.001). The frequency of vomiting and DE showed a statistically significant association (*p*<0.001).

## Discussion

This study investigated the oral and dental health disorders that can appear in patients diagnosed with eating disorders (EDs), compared with a group of healthy participants with no antecedents of EDs.

The mean age of the participants was higher than other studies that have assessed the oral health of ED patients (5,12).

The study population excluded women younger than 18 years. This condition meant that the ED had a longer time to evolve, and so sufficient time had passed since the appearance of the ED for clinical observation of any oral lesions produced by the disease.

Applying the classification of educational levels described in the (Spanish) National Health System, all participants had completed secondary education, and 68% had completed University education. This data is relevant in light of the characteristic hyper-responsibility and perfectionism presented by patients diagnosed with anorexia nervosa (18).

In the present study population, oral hygiene habits, dental check-ups, and other preventive practices were distributed between the groups without statistically significant differences, and in fact were at a higher level than usual according to the literature on the general population in Spain (19). These findings suggest that the aforementioned factors have no relationship with the pathology observed in the ED group.

The important effects of smoking on the incidence of oral mucosa lesions and as a risk factor have been described in the literature (20). Given the high frequency of mucosal lesions among ED patients, it was thought relevant to analyze the impact of smoking on oral health among the whole study population. The present work registered the number of cigarettes smoked per day (a mean of 1.68 cigarettes per day). A higher percentage of smokers were found in the EDG (33%) with significant difference in comparison with the NEDG (<20%) (*p*<0.05). These findings concur with Krug *et al.* (21) and Piper *et al.* (22), who showed that substances such as caffeine and cigarettes are used by ED patients to suppress appetite. In the present population, the relation between smoking and oral tissue lesions was clear, with a significant association between the two factors (*p*<0.05) people who smoked presented more soft tissue lesions than people who did not. However, it should be noted that practically all patients with EDG have soft tissue lesions regardless of whether they smoke or not.

DE is non-carious dental substance loss induced by direct impact of exogenous or endogenous acids and the most associated pathology with EDG. Factors that can influence the degree of erosion in ED patients include the duration and frequency of vomiting, oral hygiene habits, and diet (8). In the present study, significant differences were found between the groups, with a higher degree of DE in the EDG (*p*<0.001).

Given that the prevalence and severity of periodontal disease is directly associated with advancing age (23), it is an uncommon manifestation among patients with EDs, who are relatively young. The present study did not find differences in periodontal health between the groups. This finding agrees with Lourenço *et al.* (24), who concluded that variations in results could be due to the differences in oral hygiene maintenance within the study population. Johansson et al. (12) obtained a lower incidence of gingival bleeding in ED patients than a control group; the authors believed that this could be due to the fact that patients with EDs consider oral hygiene more important than control group subjects. In the present study it is important to emphasize that there are no differences between the groups in terms of oral hygiene habits.

Numerous studies have investigated the association between dental caries and EDs (5,12). In the present study, the decay-missing-filled (DMF) index was higher in the EDG than the NEDG, but without reaching statistical significance. The restoration index (RI) for the whole sample was 64.59%, with a higher RI in the NEDG. Jugale *et al.* (25) made a study of patients susceptible to EDs, also finding a higher caries index in comparison with healthy participants. The present results coincide with Johansson *et al.* (12), who did not find significant differences between a group of ED patients and a control group.

A strong association was found between EDs and soft tissue disorders (*p*<0.001); in fact, only one woman with ED did not present soft tissue affectation. Dry lip was present in 93.2% of the ED group but only 15.8% of the NEDG, with statistically significant difference (*p*<0.001). These results are similar to Johansson *et al.* (12), who also obtained significant differences between ED patients and control subjects without ED (*p*<0.001). Several factors such as dehydration and vomiting favor dry lip, making it more common among ED sufferers.

With regard to angular cheilitis among in the EDG, over 45% showed clinical signs of unilateral or bilateral cheilitis, while in the control group only 10.8% showed signs, with statistically significant difference between the groups (*p*<0.001). Almazrooa *et al.* (26) discovered a significant association between parafunctional lip-biting accompanied by cheilitis and ED.

It is common to find signs of erythema and ulceration among patients with EDs, particularly in the pharynx and soft palate (27). These lesions often occur among binge eaters who regurgitate afterwards. In our study, over 45% of the EDG suffered erythema in the oral mucosa and soft palate, compared with 3.3% of the NEDG (*p*<0.001). Meanwhile, significantly higher numbers (*p*<0.001) of ulcers were diagnosed in the EDG than the NEDG. This fact could be due to the possible traumatism that would occur when the vomiting with the fingers is provoked.

The literature reports that patients with EDs often present atrophic tongue and median rhomboid glossitis (28). The present study did not find a single case of atrophic tongue or median rhomboid glossitis but did find 16 cases of crenated tongue and 18 of saburral tongue. Crenated tongue was more frequent in the EDG although without significant difference between the groups. ED patients suffer more episodes of stress and anxiety that may be accompanied by tooth clenching and temporomandibular pain (29), which can be associated with tongue indentations. More saburral tongue was found among the EDG than NEDG with significant difference (*p*<0.05).

The rate of saliva flow in patients with EDs has been measured in numerous research papers (12,24,5). Our results show a significant decrease in the group of the EDG. Reductions in saliva flow are associated with a higher risk of caries, increased susceptibility to oral infection and taste alteration. Johansson *et al.* (12) registered lower values in both stimulated and non-stimulated salivary flow rates in ED patients compared with a control group, although the difference did not reach statistical significance.

When mean oral pH was compared between the groups, no difference was found. These results concur with Milosevic *et al.* (30), who, like the present work, did not find differences in pH between patients with BN and a control group. Likewise, Öhrn *et al.* (5) found no differences in pH between ED patients and healthy participants.

On the basis of the results obtained, it may be concluded that.

In the present sample, oral hygiene habits, preventive practices and dental antecedents among people with and without EDs were found to be similar. No significant differences in caries and periodontal disease were found between the groups. No differences in pH levels were found between the groups either.

The following pathologies showed significant association with EDs: dental erosion, soft tissue disorders; and reduced non-stimulated salivary flow rate were found to be more prevalent in the EDG than the NEDG.

On the basis of the results obtained, given the importance of tertiary prevention in the treatment of EDs, it is necessary to carry out oral/dental examination as soon as an ED is diagnosed with regular check-ups thereafter.

In view of these results, it would be interesting to perform a multivariate analysis with the main findings. Further prospective studies are needed with larger study populations, possibly multi-center studies that involve various clinics treating patients with EDs. In this way it would be possible to compare the results between centers to gain a clearer picture of the oral health status of patients diagnosed with ED.
